# Workplace Violence in Asian Emergency Medical Services: A Pilot Study

**DOI:** 10.3390/ijerph16203936

**Published:** 2019-10-16

**Authors:** Pei-Yu Wang, Pin-Hui Fang, Chen-Long Wu, Hsiang-Chin Hsu, Chih-Hao Lin

**Affiliations:** 1 Department of Emergency Medicine, National Cheng Kung University Hospital, College of Medicine, National Cheng Kung University, Tainan 70403, Taiwan; pat2140@gmail.com (P.-Y.W.); fph2005er@gmail.com (P.-H.F.); 505838@gmail.com (H.-C.H.); 2 Department of Occupational and Environmental Medicine, National Cheng Kung University Hospital, College of Medicine, National Cheng Kung University, Tainan 70403, Taiwan; clwu@mail.ncku.edu.tw

**Keywords:** workplace violence, emergency medical technician, emergency medical services system, Asian, Taiwan

## Abstract

Workplace violence among Asian emergency medical services (EMS) has rarely been examined. A cross-sectional, mainly descriptive study using a standardized, paper-based, self-reported questionnaire survey was conducted between August and October 2018 among emergency medical technicians (EMTs) in the Tainan City Fire Bureau, Taiwan. A total of 152 EMT-paramedics responded to the questionnaire survey, constituting an overall response rate of 96.2%. The participants were predominantly male (96.1%), college-educated (4-year bachelor’s degree) (49.3%), and middle-aged (35–44 years old) (63.8%). Among them, 113 (74.3%) and 75 (49.3%) participants had experienced verbal and physical assaults at work, respectively. Only 12 (7.9%) participants were familiar with relevant regulations or codes. The assaults predominantly occurred during evening shifts (16:00–24:00) and at the scene of the emergency. The most predominant violence perpetrators included patients, patients’ families, or patients’ friends. Nearly 10% of participants had experienced verbal assaults from hospital personnel. EMTs who encountered workplace violence rarely completed a paper report, filed for a lawsuit, or sought a psychiatric consultation. Fifty-eight (38.2%) and 16 (10.5%) participants were victims of frequent (at least once every 3 months) verbal and physical forms of violence, respectively; however, no statistically significant association was observed in terms of EMT gender, age, working years, education level, or the number of EMS deployments per month. The prevalence of workplace violence among Asian EMS is considerable and is comparable to that in Western countries. Strategies to prevent workplace violence should be tailored to local practice and effectively implemented.

## 1. Introduction

Workplace violence is a global issue. Workplace violence is defined as any act or threat of psychological or physical violence, harassment, intimidation, or other threatening disruptive behavior that occurs at work [1]. Compared to other occupations, emergency medical services (EMS) have a high risk of workplace violence [2,3,4]. Emergency medical technicians (EMTs) usually respond to medical emergencies and access unfamiliar patients in stressful environments. EMTs have an occupational fatality rate comparable to those of police and firefighters and a nonfatal injury rate that is much higher than the average for all workers [5,6].

A recent literature review found that the frequency of verbal violence toward EMS providers was about 21%–82%, while physical violence was reported to occur in about 13%–79% of cases [3]. More than 80% of EMTs in Australia [7] and Sweden [8] have experienced workplace violence during the past 12 months. Approximately 61%–93% of EMTs in the United States have been physically assaulted during their careers [2,9,10]. The characteristics of violence victims are inconsistent in previous studies [3]. One study showed that EMT gender, work experience, and professional level did not differ significantly in the face of workplace violence [11]. Some studies revealed that the less experienced were more susceptible to violence [12], and female EMTs were more likely to encounter sexual assaults [13]. However, other studies showed that veteran paramedics [13] and male EMS practitioners [14] encountered violence more often.

Workplace violence jeopardizes self-esteem and quality of work [4,6]. Violence and assaults at work may lead to psychological and physical sequalae [15,16]. EMTs confronted by violent assaults may experience higher stress levels, physical injury, decreased job satisfaction, anxiety, avoidance behavior, negative impact on personal relationships, and even suicide [3,17]. Workplace violence impairs the relationship between the paramedic and the patient and causes a drop in quality of care [8]. Preventing workplace violence toward EMS personnel is important not only for EMS practitioners but also for patients in need of prehospital medical care [3,4,8].

The factors affecting workplace violence toward EMS personnel include gender, society, race, economic status, religion, and/or culture [8,11,18,19,20,21,22]. People of Asian background are often described as having a less emotionally active or aggressive personality [23]; subsequently, workplace violence in Asian communities might be neglected and underreported. While the prevalence of verbal and physical forms of workplace violence among Taiwanese workers over the past 12 months were estimated to be 8.3% and 1.2%, respectively [24], to date it appears that workplace violence in Asian EMS has rarely been examined in the literature. Gaining an understanding of the prevalence and other information about workplace violence is a fundamental step to build up a safe occupational environment. The knowledge of EMT’s reactions toward assaults could contribute to the improvement of training programs, organization culture, and even public policymaking. Therefore, this study aims to identify the occupational risks of physical and verbal violence in Taiwanese EMS. Potential solutions to prevent workplace violence in EMS are also explored.

## 2. Methods

### 2.1. Study Setting and Design

This pilot study was a cross-sectional, mainly descriptive survey study using a questionnaire survey. It was completed between August and October 2018 in Tainan City, Taiwan. The governmental EMS in Taiwan are all fire-based service. There are three levels of care providers (EMT-basic, EMT-intermediate, EMT-paramedic) and the services are generally free of charge. Tainan City has a population of 1.9 million and covers an area of 2192 km^2^. There are 55 fire stations in Tainan City. The annual EMS call volume in Tainan City was 95,310 in 2018, which is equivalent to 13.8 calls per 100,000 population per day. During the study period, there were 1024 EMTs (aged 27.0–55.0 years, mean 38.2 years; 946 (92.4%) of whom were male) affiliated with the Tainan City Government Fire Bureau. Among them, 58 (5.7%) were EMT-basic, 810 (79.1%) were EMT-intermediate, and 156 (15.2%) were EMT-paramedic.

The survey was conducted during a three-session, continuing education course for EMT-paramedics. All of the survey participants were EMT-paramedics who were affiliated with the Tainan City Government Fire Bureau. Participation in the survey was voluntary and confidential. A 30-min debriefing session to promote the awareness of occupational safety and workplace violence was delivered, following which an anonymous questionnaire was distributed, and it was collected 30 min later.

### 2.2. Survey Questionnaire

The survey took the form of a standardized, paper-based, self-reported questionnaire. The questionnaire was modified partially by reference to the Workplace Violence in the Health Sector Country Case Studies Research Instruments Survey Questionnaire [25] as well as to questionnaires that had been used in relevant studies [22,26].

In our questionnaire survey, verbal violence was defined as using offensive language, yelling or screaming with the intent to offend or frighten. Verbal violence could be delivered through the phone. Physical violence was defined as a physical assault or any attempt at a physical attack. Physical assaults include behaviors, such as punching, slapping, kicking, or using a weapon or other objects with intent to cause bodily harm. In this pilot study, we did not address sexual harassment or gender-related mistreatment, which could be a form of workplace violence.

The survey questionnaire had three parts: participants’ information (Part I), experience of workplace violence (Part II), and strategies to prevent workplace violence (Part III). Part I included demographic information about the participants (such as gender, age, and education level), EMS career experience (such as length of EMS career, working hours per month, and the number of EMS deployments per month), and knowledge of relevant regulations or codes regarding workplace violence. Part I of the survey mainly used closed-ended, multiple-choice, and single-answer questions.

If a participant had experienced workplace violence, then completed Part II of the survey. Part II included questions regarding the experience of verbal or physical violence in the workplace. It allowed questions of the perpetrator types (such as patients, families or friends of patients, bystanders, or colleagues), and the time and location of workplace violence (for example, at the emergency scene, during transportation to the hospital, at the hospital, and/or at the fire station). It also included the response to workplace violence (for example, trying to comfort the perpetrators, discussion with colleagues, completing a paper report, applying for a job transfer, seeking psychiatric consultation, and/or reporting the incident to the news media) and asked about the personal impact of workplace violence (such as personal mood, professional performance, interpersonal relationships, and family relationships). Part II of the survey mainly used closed-ended, multiple-choice, and multiple answer questions.

All participants participated in Part III of the survey. Part III identified six domains of strategies to prevent workplace violence. These included knowledge and training (such as education, simulation training, and consultations for emotional control), equipment and resources (such as the use of chemical or physical restraints, video recording, self-defense techniques, and bullet-proof helmets) and systems (such as reporting system, police activation system, and patient records). In addition, it addressed policy considerations (such as EMS agency policy, advocation of antiviolence policy, and use of internet resources and social media), situation awareness (such as keeping a safe distance, improving situation awareness, self-protection strategies, and removing dangerous materials), and security (such as warning systems, continuing video recording during the time of duty, and police escorts). In Part III, a five-point Likert scale (strongly disagree, disagree, neutral, agree and strongly agree) was used. The items of “agree” and “strongly agree” were considered a positive response to the question, while the other items were not.

The English version of the questionnaire used in this study is provided in Appendix A.

### 2.3. Data Analysis

Data were entered into an Excel (Microsoft, OVS-ES, 2016) database, and statistical analyses were performed using SPSS 17.0 software (SPSS Inc., Chicago, IL, USA). Descriptive proportions were used for the categorical variables. The chi-squared and Fisher’s exact tests were applied to analyze the differences for the nominal variables, as applicable.

We assumed that the frequency of workplace violence could reveal more insights. EMTs who experienced verbal or physical assaults at least once every 3 months were defined as victims of frequent workplace violence. Multivariate logistic regression analyses were performed to identify the risk factors for frequent workplace violence using odds ratios (ORs) and 95% confidence intervals (CIs). A two-tailed *p*-value of less than 0.05 indicated statistical significance.

### 2.4. Ethical Considerations

The data was de-identified, allowing participants to remain anonymous throughout the research. The study protocol was reviewed and approved by the Institutional Review Board of National Cheng Kung University Hospital (B-ER-107-338).

## 3. Results

### 3.1. Characteristics of Participants

In this survey, of 156 EMT-paramedics (149 (95.5%) of whom were male) who completed the continuing education courses between August and October 2018, 152 responded to the questionnaire. The overall response rate was 96.2%. Table 1 describes the basic demographic information of the participants.

The participants were predominantly male (96.1%), college-educated (4-year bachelor’s degree) (49.3%), and middle-aged (35–44 years old) (63.8%). Totals of 113 (74.3%) and 75 (49.3%) respondents reported that they had ever experienced verbal and physical forms of workplace violence, respectively. In addition, more than 80% of participants reported that their colleagues had experienced workplace violence. Only 12 (7.9%) participants were familiar with the regulations regarding workplace violence, with more than half of the respondents did not know that such regulations even existed.

### 3.2. Frequency of Workplace Violence 

Table 2 describes the frequency of verbal and physical assaults that the participants experienced during work. Participants generally experienced verbal assaults more frequently than physical assaults.

### 3.3. Pattern of Workplace Violence 

Table 3 summarizes the patterns of workplace violence. Violence perpetrators were predominately male. Either verbal assaults (47.8%) or physical assaults (48.0%) were more likely to occur during evening shifts (16:00–24:00). The assaults predominantly occurred at the emergency scene. The most predominant violence perpetrators included patients, patients’ families, or patients’ friends. Among patients who were treated by EMTs, patients with substance abuse or alcohol intoxication were most likely to assault, either verbally or physically.

### 3.4. Response to Workplace Violence 

Table 4 describes the response to and impact of encountering workplace violence for the EMTs in this study. Most of the participants’ immediate reaction was taking a deep breath and trying to manage the patient while they encountered verbal (68.1%) or physical violence (58.7%). EMTs were more likely to file a lawsuit when they experienced physical violence compared to verbal violence (14.7% vs. 3.5%, *p* = 0.006). None of the 113 participants who experienced verbal assaults and only 1 (1.3%) of the 75 participants who experienced physical assaults had sought a psychiatric consultation. Workplace violence may affect personal mood in more than 80% of the participants and professional performance in nearly half of the EMTs.

Few participants reported the occurrence of verbal violence (8.8%) or physical violence (22.7%) to their superiors at the fire bureau. The behavior of reporting workplace violence to the superiors is of interest; however, the bivariate analysis found no significant association with respect to EMT age, working years, education level, or the number of EMS deployments per month (Appendix B).

### 3.5. Risk Factors of Frequent Workplace Violence

A total of 58 (38.2%) and 16 (10.5%) participants were victims of frequent (that is, at least once every 3 months) verbal and physical forms of violence, respectively. Table 5 shows the bivariate and multivariate logistic regression analysis of the risks for frequent verbal or physical violence. Regarding frequent workplace violence, no statistically significant association was observed in terms of gender, age, working years, education level, or the number of EMS deployments per month.

### 3.6. Workplace Violence Prevention

Appendix C shows the participants’ perceptions of strategies to prevent workplace violence. Regarding the domain of knowledge and training, more than 70% of participants believed that anti-violence law enforcement could be effective in preventing workplace violence. Video-recording devices were the most feasible equipment to prevent either verbal (83.6%) or physical violence (84.2%). Nearly 80% of participants indicated that a registry of patients with a known history of violent assaults or substance abuse could lessen workplace violence. Nearly 90% of participants said that maintaining a safe distance and improving situation sensitivity could be useful.

## 4. Discussion

People of Asian background are often described as having a tendency to avoid conflicts and confrontations [23]; however, our study shows that workplace violence toward EMTs is common in Asian communities. The prevalence of workplace violence in the Taiwan EMS was comparable to that in Western countries [2,3,7,8,9,10]. The prevelance of verbal violence and physical violence among EMS could be at least 8 times and 40 times greater, respectively, than that of overall workers in Taiwan [24]. Very few EMTs were familiar with the regulations or codes regarding workplace violence. Workplace violence across Asian EMS deserves further research and in-depth discussion.

The association of EMT gender and workplace violence risks is controversial [2,13,26,27,28]. In our study, 109 (74.7%) of 146 male participants and 4 (66.7%) of 6 female participants experienced verbal assaults at work (*p* = 0.646). In addition, 74 (50.7%) of 146 male participants and 1 (16.7%) of 6 female participants experienced physical assaults (*p* = 0.210). The percentage of male EMTs who had experienced workplace violence, either in the form of verbal or physical assaults, was higher than that of female EMTs. However, with respect to frequent workplace violence (at least once every 3 months), we did not find any significant association in terms of EMT gender, age, working years, education level, or the number of EMS deployments per month. Other factors, such as personality traits, coping styles, attitudes or participating in violence prevention training, could be associated with risks for work-related threats [29]. However, we were unable to explore the association of personality characteristics or communication skills with the risks of workplace violence in this study.

Patients, especially those who have cognitive dysfunction [30,31,32], are some of the most common perpetrators of violence [26]. EMTs should be extremely cautious when handling patients with substance abuse or alcohol intoxication. We also found that patients’ families or friends were perpetrators more often. Patients’ families or friends may request a rush transfer to a hospital, rather than proper and necessary medical care in the prehospital setting prior to transport [4]. Managing emotionally agitated patients or families can cause anxiety in even the most seasoned EMTs. Using verbal de-escalation and certain coping techniques when dealing with people with aggression or agitation appears to be accepted as good clinical practice [33]. EMTs should also monitor their own emotional and physiological response so as to remain calm and alert at an emergency scene.

Many Asian EMS are public sectors and the services are generally free of charge to users [34]. Public sector workers, compared with private sector employees, could have higher risks of workplace violence and higher levels of client-related burnout [24]. Public perception of EMT professionalism may be underrecognized and should be promoted in Asian communities. However, training on interpersonal skills and emotional intelligence in a professional context is often lacking within Asian EMS system. Simulation training and other education modules could enhance knowledge of workplace violence [35]. We strongly recommend that communication skills and clinical empathy should be integrated into EMT education programs [36,37].

Verbal assaults were the most common form of workplace violence [2,7]. Verbal violence, especially in the fields of emergency and intensive care medicine, is a known hazard for healthcare-related personnel [38]. Verbal assaults toward prehospital care personnel are not uncommon and occur mostly at the emergency scene [39,40,41]. However, our study revealed that verbal assaults toward EMTs could occur in hospitals and could be delivered by hospital personnel. Overcrowding in emergency departments has become a public health problem worldwide in the last decade. Heavy workload and emergency department overcrowding could render emergency physicians and nurses to frustration and feelings of burnout [42,43]. When EMTs transport patients to the emergency departments, the impression of increasing emergency department workload may generate conflicts between hospital personnel and EMS providers. Beyond the difficulties arising from ambulance diversion practices, many EMTs find themselves detained in emergency departments for prolonged periods, unable to transfer care of their transported patients to hospital staff [44,45]. Building a collaborative approach and developing a mutual understanding environment, especially among EMTs and emergency medicine personnel, should be emphasized in regional emergency care systems.

EMTs who encountered workplace violence rarely completed a paper report, verbally reported the incident to their superior, or filed for a lawsuit. The silence of Asian EMTs toward workplace violence is noteworthy. It is beyond the scope of this study to thoroughly explore the reasons of silence; however, potential causes of silence at the collective level and on the organization-level factors were identified [46,47]. Our study showed that most EMTs were unaware of regulations regarding workplace violence. Voice could be curtailed if EMTs do not feel that their reports will be taken fairly and acted upon [48]. EMTs may remain quiet when they think that they may suffer unwanted consequences [48]. The feeling of futility to speak up is a key determinant of acquiescence [49]. Another important judgment could be the extent to which it is safe or supported to engage in actions against workplace violence [50,51]. 

The hierarchical and “masculine” culture of EMS might impede the pursuit of legal or administrative justification or even professional assistance, such as psychiatric consultation [14,17,52]. Some EMTs may even have a perception that experiencing violence is necessarily part of the job [4,22]. In our study, more than 80% of EMTs reported that their moods were obviously affected by workplace violence. Either verbal or physical assaults could impair professional performance in nearly half of the EMTs. Therefore, regulations against workplace violence should be developed not only for the mental health of EMTs but also for the quality of patient care. Awareness of occupational safety should be promoted among Asian EMS as well. Certain tools could be utilized to evaluate workplace violence risks and prevention modules [53]. Reporting might be valuable and contribute to improvement of the situation. A proper reporting protocol with considerations of protecting personal information should also be provided when EMTs encounter assaults [16]. Strategies to prevent workplace violence in EMS systems should be tailored to local practice.

## 5. Limitations

Our study had several limitations. First, our study used a self-administered questionnaire survey; thus, the accuracy of the survey results could be biased due to cultural and personal factors. Nevertheless, the anonymity of the survey may accurately gauge the true experience of EMTs and reflect a certain reality. Second, we did not explore several potentially important factors. We did not evaluate the personality characteristics of the EMTs in this study, which could be valuable for the analysis of behavior patterns and risks of workplace violence. The work culture in each individual fire station and EMS team could vary and may deserve investigation as well. In this pilot study, we were unable to investigate sexual harassment or gender-related mistreatment, which is a form of workplace violence. Third, the obvious disproportion of EMT gender in our study, which was inherent in many Asian EMS as well, may hinder further analysis of workplace violence regarding the role of gender. Finally, since our study only assessed EMT-paramedics in one single EMS system, further research might be needed to validate the generalizability and applicability of our study results.

## 6. Conclusions

The prevalence of workplace violence in Asian EMS is considerable and is comparable to that in Western countries [2,3,7,8,9,10]. Many EMTs are unaware of regulations regarding workplace violence. EMTs rarely report workplace violence to their superiors. EMT training on interpersonal skills and emotional intelligence needs to be strengthened. Public perception of EMT professionalism could be promoted in Asian communities. A mutual understanding environment between EMTs and emergency medicine personnel should be developed. The identification of risk factors for workplace violence could provide important guidance for policymaking and education programs. The silence of Asian EMTs toward workplace violence warrants further exploration. Workplace violence prevention should be tailored to local practice and effectively implemented.

## Figures and Tables

**Table 1:** Personal characteristics and work experience of the study participants.

	Participants N = 152 (%)
**Gender (Male)**	146 (96.1)
**Age (Years)**	
20–34	42 (27.6)
35–44	97 (63.8)
45–55	13 (8.6)
**Work experience (Years)**	
≤5	1 (0.7)
6–10	25 (16.4)
11–15	57 (37.5)
≥16	69 (45.4)
**Education level**	
2-year college	48 (31.6)
4-year college	75 (49.3)
Master’s degree	29 (19.1)
**Number of EMS deployments per month**	
≤20	56 (36.8)
21–50	37 (24.5)
50–100	36 (23.8)
≥101	23 (15.2)
Have you ever experienced verbal assaults during work? (Yes)	113 (74.3)
Have you ever experienced physical assaults during work? (Yes)	75 (49.3)
Have your colleagues ever experienced verbal assaults during work? (Yes)	123 (80.9)
Have your colleagues ever experienced physical assaults during work? (Yes)	123 (80.9)
Is verbal assault a serious problem in your work environment? (Yes)	92 (60.5)
Is physical assault a serious problem in your work environment? (Yes)	72 (47.4)
**Perceptions of occupational violence regulations (Single answer) (N = 151)**	
My department has relevant regulations, and I understand the relevant content	12 (7.9)
My department has relevant regulations, but I am unsure about the relevant content	15 (9.9)
My department does not have such regulations	78 (51.7)
I am unsure whether my department has such regulations	46 (30.5)

**Table 2:** Frequency of workplace violence.

	Verbal Assaults N = 113 (%)	Physical Assaults N = 75 (%)	*p*-Value
**How many times did you experience violent assaults during work in the past ten years?**			<0.001
≤10	76 (67.3)	71 (94.7)	
11–20	12 (10.6)	2 (2.7)	
21–50	7 (6.2)	1 (1.3)	
≥51	18 (15.9)	1 (1.3)	
**How many times did you experience violent assaults during work in the past one year?**			0.027
≤10	100 (88.5)	74 (98.7)	
11–20	7 (6.2)	0	
21–50	3 (2.7)	1 (1.3)	
≥51	3 (2.7)	0	
**How many times did you experience violent assaults during work in the past one month?**			0.836
≤10	112 (99.1)	75 (100)	
11–20	1 (0.9)	0	
21–50	0	0	
≥51	0	0	
**How frequently did you experience violent assaults?**			<0.001
Every week	13 (11.5)	6 (8.0)	
Every month	24 (21.2)	5 (6.7)	
Every 3 months	21 (18.6)	5 (6.7)	
Every year	44 (38.9)	59 (78.7)	

**Table 3:** Patterns of workplace violence.

	Verbal Assaults N = 113 (%)	Physical Assaults N = 75 (%)	*p*-Value
**The gender of violence perpetrators (Multiple answers)**			
Male	112 (99.1)	71 (94.7)	0.083
Female	49 (43.4)	29 (38.7)	0.522
**During which time are you likely to experience violent assaults? (Multiple answers)**			
Day shift (08:00–16:00)	23 (20.4)	10 (13.3)	0.127
Evening shift (16:00–24:00)	54 (47.8)	36 (48.0)	0.977
Night shift (00:00–08:00)	31 (27.4)	25 (33.3)	0.386
**At which location did you ever experience violence assaults? (Multiple answers)**			
At the scene	99 (87.6)	66 (88.0)	0.936
During transportation to the hospital	53 (46.9)	32 (42.7)	0.567
At the hospital	19 (16.8)	15 (20.0)	0.578
At the fire station	37 (32.7)	5 (6.7)	<0.001
**By whom have you ever been assaulted? (Multiple answers)**			
Patients	90 (79.6)	67 (89.3)	0.079
Patients’ families	93 (82.3)	42 (56.0)	<0.001
Patients’ friends	80 (70.8)	38 (50.7)	0.005
Bystanders	58 (51.3)	19 (25.3)	<0.001
Fire bureau colleagues	26 (23.0)	6 (8.0)	0.007
Other governmental officers (such as policepersons or traffic light regulators)	24 (21.2)	3 (4.0)	0.001
Physicians	10 (8.8)	0	0.007
Nurses	13 (11.5)	0	0.002
Hospital administrators	16 (14.2)	1 (1.3)	0.003
**By which kind of patients have you ever been assaulted? (Multiple answers)**			
Traumatic patients with clear consciousness	48 (42.5)	26 (34.7)	0.283
Traumatic patients with unclear consciousness that was mainly due to trauma	21 (18.6)	21 (28.0)	0.129
Traumatic patients with unclear consciousness that was mainly due to alcohol intoxication or drug abuse	79 (69.9)	50 (66.7)	0.638
Nontraumatic patients with clear consciousness	44 (38.9)	27 (36.0)	0.684
Nontraumatic patients with unclear consciousness that was mainly due to illness	30 (26.5)	19 (25.3)	0.852
Nontraumatic patients with unclear consciousness that was mainly due to alcohol intoxication or drug abuse	81 (71.7)	47 (62.7)	0.194
Pregnant patients	4 (3.5)	1 (1.3)	0.357
Pediatric patients	4 (3.5)	1 (1.3)	0.357

**Table 4:** Responses to workplace violence.

	Verbal Assaults N = 113 (%)	Physical Assaults N = 75 (%)	*p*-Value
**How would you react immediately (Multiple answers)**			
No response at all	31 (27.4)	10 (13.3)	0.022
Pretend nothing happened	15 (13.3)	4 (5.3)	0.077
Comfort the perpetrators	62 (54.9)	33 (44.0)	0.144
Take a deep breath and manage the patients first	77 (68.1)	44 (58.7)	0.184
Fight back immediately	22 (19.5)	20 (26.7)	0.245
Discuss with colleagues	18 (15.9)	15 (20.0)	0.472
**What would you do when you return to fire departments? (Multiple answers)**			
Complete a paper report at the fire bureau	0	3 (4.0)	0.062
Apply for a job transition	1 (0.9)	1 (1.3)	0.379
Seek psychiatric consultation	0	1 (1.3)	0.086
Verbally report to superiors at the fire bureau	10 (8.8)	17 (22.7)	0.008
**Would you report workplace violence to news or social media? (Multiple answers)**			
Report to news media, such as television	0	3 (4.0)	0.062
Report to social media, such as Facebook	6 (5.3)	2 (2.7)	0.379
**Would you take legal action? (Multiple answers)**			
Report to the police	27 (23.9)	33 (44.0)	0.004
File for a lawsuit	4 (3.5)	11 (14.7)	0.006
Request an apology from the perpetrator	5 (4.4)	9 (12.0)	0.052
Request financial compensation	1 (0.9)	8 (10.7)	0.002
**How did violence assaults affect you? (Multiple answers)**			
Personal mood	95 (84.1)	68 (90.7)	0.192
Professional performance	49 (43.4)	38 (50.7)	0.353
Interpersonal relationship	11 (9.7)	9 (12.0)	0.621
Family relationship	9 (8.0)	8 (10.7)	0.527
No impact	13 (11.5)	7 (9.3)	0.636

**Table 5:** Regression analysis of risks for frequent (at least once every 3 months) verbal or physical violence.

	Frequent Verbal Violence (N = 58)		Frequent Physical Violence (N = 16)	
	ODs (95% CIs)	aODs (95% CIs)	ODs (95% CIs)	aODs (95% CIs)
**Gender**				
Female	Ref.	Ref.	Ref.	Ref.
Male	0.60 (0.12–3.10)	0.60 (0.10–3.58)	0.57 (0.06–5.23)	0.76 (0.06–9.34)
**Age (years)**				
20–34	Ref.	Ref.	Ref.	Ref.
35–44	0.65 (0.31–1.37)	0.71 (0.27–1.81)	0.54 (0.17–1.66)	0.72 (0.17–2.98)
45–55	0.76 (0.21–2.70)	1.38 (0.30–6.41)	1.09 (0.19–6.20)	3.55 (0.36–34.61)
**Working years**				
≤10	Ref.	Ref.	Ref.	Ref.
11–15	1.07 (0.42–2.72)	1.30 (0.46–3.69)	1.25 (0.30–5.16)	1.29 (0.26–6.44)
≥16	0.64 (0.25–1.61)	0.77 (0.24–2.46)	0.60 (0.13–2.71)	0.61 (0.09–4.11)
**Education level**				
2-year college	Ref.	Ref.	Ref.	Ref.
4-year college	0.90 (0.42–1.92)	1.21 (0.52–2.83)	0.40 (0.11–1.50)	0.49 (0.11–2.08)
Master’s degree	1.46 (0.58–3.68)	2.32 (0.78–6.90)	1.75 (0.51–6.03)	2.77 (0.58–13.30)
**EMS deployments per month**				
≤20	Ref.	Ref.	Ref.	Ref.
21–50	1.29 (0.54–3.07)	1.13 (0.44–2.86)	1.59 (0.43–5.94)	1.26 (0.28–5.59)
51–100	1.51 (0.63–3.59)	2.04 (0.77–5.35)	1.27 (0.32–5.10)	1.95 (0.39–9.70)
≥101	1.94 (0.72–5.22)	2.58 (0.89–7.51)	0.97 (0.17–5.41)	1.35 (0.21–8.64)

Abbreviations: ODs, odds ratios; aODs, adjusted odds ratios.

## Data Availability

The datasets used or analyzed during the current study are available from the corresponding author on reasonable request.

## Appendix A. Survey Questionnaire for Workplace Violence toward Emergency Medical Technicians

Part I. Basic Information

A1. Gender

  □ Male □ Female

A2. Age (years)

  □ less than 20 □ 20–34 □ 35–44 □ 45–54 □ 55 and above

A3. Work experience (Year)

  □ less than 1 year □ 1–5 □ 6–10 □ 11–15 □ 16 years or more

A4. Educational level

  □ Elementary school □ Junior high school □ 2-year-college □ 4-year college

  □ Master’s degree

A5. Work hours of filed deployment per month:    hours

A6. The number of deployments per month

  □ less than 10 times □ 11–20 times □ 21–50 times □ 51–100 times

  □ more than 100 times

A7. Perceptions of the regulations regarding occupational violence.

  □ My department has regulations regarding occupational violence, and I clearly understand the relevant content

  □ My department has regulations regarding occupational violence, but I am not sure about the relevant content

  □ I do not think my department has regulations regarding occupational violence

  □ I am unsure whether or not my department has regulations regarding occupational violence

A8. Is verbal assault a serious problem in your work environment?

  □ very serious □ serious □ neutral □ not serious □ absolutely not

A9. Is physical assault a serious problem in your work environment?

  □ very serious □ serious □ neutral □ not serious □ absolutely not

Part II. Patterns of Workplace Violence (Verbal Assaults)

Verbal violence was defined as using offensive language, yelling or screaming with the intent to offend or frighten. Verbal assaults could be delivered through the phone.

B1. Have your colleagues ever experienced verbal assaults during work?

  □   Yes □ No

B2. Have you ever experienced verbal assaults during work?

  □ Yes (If yes, please go to B3)

  □ No (If no, please go to B14)

B3. How many times did you experience verbal violence during work?

B3.1 In the past ten years?

  □ 0–5 times □ 6–10 times □ 11–20 times □ 21–50 times □ more than 50

B3.2 In the past one year?

  □ 0–5 times □ 6–10 times □ 11–20 times □ 21–50 times □ more than 50

B3.3 In the past one month?

  □ 0-5 times □ 6–10 times □ 11–20 times □ 21–50 times □ more than 50

B4. How frequently did you experience verbal violence?

  □ At least once per week □ At least once per month □ At least once per three months

  □ At least once per year

B5. What are the gender(s) of violence perpetrators? (can choose more than one answer)

  □ Male □ Female

B6. During what time are you likely to experience verbal violence? (can choose more than one answer)

  □ Day shift (08:01–16:00) □ evening shift (16:01–24:00) □ night shift (00:00–08:00)

B7. At which location did you ever experience verbal violence? (can choose more than one answer)

  □ In the field scene of patients □ During the transportation of patients to hospitals

  □ In hospitals □ In fire stations

B8. At which location are you most likely to experience verbal violence?

  □ At the emergency scene of patients □ During transportation of patients to the hospitals

B9. By whom have you ever been verbally assaulted? (can choose more than one answer)

  □ Patients □ Families of patients □ Friends of patients □ Bystanders

  □ Colleagues

  □ Other governmental officers (such as policepersons, traffic lights regulators, etc.)

  □ Hospital administrators (□ Physician/□ Nurse/□ other:___________)

B10. Who is the most likely person to verbally assault you?

  □ Patient □ Patient’s families □ Patient’s friends □ Bystanders □ Colleagues

  □ Other governmental officers (such as policepersons, traffic lights regulators, etc.)

  □ Hospital administrators (□ Physician/□ Nurse/□ other:___________)

B11. By which kind of patients have you ever been verbally assaulted? (can choose more than one answer)

  □ Traumatic patients with clear consciousness

  □ Traumatic patients with unclear consciousness due to trauma

  □ Traumatic patients with unclear consciousness due to alcohol intoxication or drug abuse

  □ Nontraumatic patients with clear consciousness

  □ Nontraumatic patients with unclear consciousness due to illness

  □ Nontraumatic patients with unclear consciousness due to alcohol intoxication or drug abuse

  □ Pregnant patients

  □ Pediatric patients

B12. What do you do when you experience verbal violence? (can choose more than one answer)

  □ No response at all      □ Try to pretend nothing happened

  □ Try to comfort the perpetrators   □ Discuss with colleagues

  □ Fight back immediately    □ Hold breath and manage the patients first

  □ Request an apology form the perpetrator □ Report to superiors at the fire bureau

  □ Report to policepersons     □ Seek psychiatric consultation

  □ Apply for a job transition   □ Report on social media, such as Facebook

  □ Complete a paper report     □ Report to news media, such as TV

  □ File for a law suit         □ Request compensation from the perpetrator

  □ Others:___________

B13. What impact did verbal violence have on you? (can choose more than one answer)

  □ Personal mood □ Interpersonal relationship □ Family relationship

  □ Professional performance □ No impact

Part II. Patterns of Workplace Violence (Physical Assaults)

Physical violence was defined as a physical attack or any attempt at a physical attack. Physical assaults may include behaviors such as punching, slapping, kicking, or using a weapon or other objects with the intent of causing bodily harm.

B1. Have your colleagues ever experienced physical assaults during work?

  □ Yes   □ No

B2. Have you ever experienced physical assaults during work?

  □ Yes (If yes, please go to B3)

  □ No (If no, please go to B14)

B3. How many times did you experience physical violence during work?

B3.1 In the past ten years?

  □ 0–5 times □ 6–10 times □ 11–20 times □ 21–50 times □ more than 50

B3.2 In the past one year?

  □ 0–5 times □ 6–10 times □ 11–20 times □ 21–50 times □ more than 50

B3.3 In the past one month?

  □ 0–5 times □ 6–10 times □ 11–20 times □ 21–50 times □ more than 50

B4. How frequently did you experience physical violence?

  □ At least once per week □ At least once per month □ At least once per three months

  □ At least once per year

B5. What are the gender(s) of violence perpetrators? (can choose more than one answer)

  □ Male □ Female

B6. During what time are you likely to experience physical violence? (can choose more than one answer)

  □ Day shift (08:01–16:00) □ evening shift (16:01–24:00) □ night shift (00:00–08:00)

B7. At which location did you ever experience physical violence? (can choose more than one answer)

  □ In the field scene of patients □ During the transportation of patients to hospitals

  □ In hospitals □ In fire stations

B8. At which location are you most likely to experience physical violence?

  □ At the emergency scene of patients □ During transportation of patients to the hospitals

B9. By whom have you ever been physically assaulted? (can choose more than one answer)

  □ Patients □ Families of patients □ Friends of patients □ Bystanders

  □ Colleagues

  □ Other governmental officers (such as policepersons, traffic lights regulators, etc.)

  □ Hospital administrators (□ Physician/□ Nurse/□ other:___________)

B10. Who is the most likely person to physically assault you?

  □ Patient □ Patient’s families □ Patient’s friends □ Bystanders □ Colleagues

  □ Other governmental officers (such as policepersons, traffic lights regulators, etc.)

  □ Hospital administrators (□ Physician/□ Nurse/□ other:___________)

B11. By which kind of patients have you ever been physically assaulted? (can choose more than one answer)

  □ Traumatic patients with clear consciousness

  □ Traumatic patients with unclear consciousness due to trauma

  □ Traumatic patients with unclear consciousness due to alcohol intoxication or drug abuse

  □ Nontraumatic patients with clear consciousness

  □ Nontraumatic patients with unclear consciousness due to illness

  □ Nontraumatic patients with unclear consciousness due to alcohol intoxication or drug abuse

  □ Pregnant patients

  □ Pediatric patients

B12. What do you do when you experience physical violence? (can choose more than one answer)

  □ No response at all □ Try to pretend nothing happened

  □ Try to comfort the perpetrators □ Discuss with colleagues

  □ Fight back immediately □ Hold breath and manage the patients first

  □ Request an apology form the perpetrator □ Report to superiors at the fire bureau

  □ Report to policepersons        □ Seek psychiatric consultation

  □ Apply for a job transition   □ Report on social media, such as Facebook

  □ Complete a paper report    □ Report to news media, such as TV

  □ File for a lawsuit         □ Request compensation from the perpetrator

  □ Others:___________

B13. What impact did physical violence have on you? (can choose more than one answer)

  □ Personal mood □ Interpersonal relationship □ Family relationship

  □ Professional performance □ No impact

Part III. Strategies to Prevent Workplace Violence (Verbal Assaults)

B14. What kind of strategy may prevent verbal violence during work?

Knowledge/training

	strongly disagree	disagree	neutral	agree	strongly agree
1. Educational lecture	□	□	□	□	□
2. Simulation training	□	□	□	□	□
3. Emotion control lecture/consultation	□	□	□	□	□
4. Antiviolence law	□	□	□	□	□
5. Other:___________					

Equipment/resource

	strongly disagree	disagree	neutral	agree	strongly agree
1. Chemical or physical restraints	□	□	□	□	□
2. Video recording	□	□	□	□	□
3. Self-defense technique (i.e., baton)	□	□	□	□	□
4. Bullet-proof vest/helmet	□	□	□	□	□
5. Other:___________					

System

	strongly disagree	disagree	neutral	agree	strongly agree
1. Reporting warning system	□	□	□	□	□
2. Connecting system between police and firefighter	□	□	□	□	□
3. Violent patient profile	□	□	□	□	□
4. Substance abuse record	□	□	□	□	□
5. Other:___________					

Policy/Society

	strongly disagree	disagree	neutral	agree	strongly agree
1. EMS agency policy	□	□	□	□	□
2. Advertisement of occupational violence	□	□	□	□	□
3. Standardization of process	□	□	□	□	□
4. Internet resource and social media	□	□	□	□	□
5. Other:___________					

Situation awareness

	strongly disagree	disagree	neutral	agree	strongly agree
1. Safe distance	□	□	□	□	□
2. Keep awareness	□	□	□	□	□
3. Protection strategy	□	□	□	□	□
4. Remove dangerous materials	□	□	□	□	□
5. Other:___________					

Security

	strongly disagree	disagree	neutral	agree	strongly agree
1. Reporting system	□	□	□	□	□
2. Video recording while on duty	□	□	□	□	□
3. Sufficient equipment for protection	□	□	□	□	□
4. Having policepersons escort	□	□	□	□	□
5. Other:___________					

Part III. Strategies to Prevent Workplace Violence (Physical Assaults)

B14. What kind of strategy may prevent verbal violence during work?

Knowledge/training

	strongly disagree	disagree	neutral	agree	strongly agree
1. Educational lecture	□	□	□	□	□
2. Simulation training	□	□	□	□	□
3. Emotion control lecture/consultation	□	□	□	□	□
4. Antiviolence law	□	□	□	□	□
5. Other:___________					

Equipment/resource

	strongly disagree	disagree	neutral	agree	strongly agree
1. Chemical or physical restraints	□	□	□	□	□
2. Video recording	□	□	□	□	□
3. Self-defense technique (i.e., baton)	□	□	□	□	□
4. Bullet-proof vest/helmet	□	□	□	□	□
5. Other:___________					

System

	strongly disagree	disagree	neutral	agree	strongly agree
1. Reporting warning system	□	□	□	□	□
2. Connecting system between police and firefighter	□	□	□	□	□
3. Violent patient profile	□	□	□	□	□
4. Substance abuse record	□	□	□	□	□
5. Other:___________					

Policy/Society

	strongly disagree	disagree	neutral	agree	strongly agree
1. EMS agency policy	□	□	□	□	□
2. Advertisement of occupational violence	□	□	□	□	□
3. Standardization of process	□	□	□	□	□
4. Internet resource and social media	□	□	□	□	□
5. Other:___________					

Situation awareness

	strongly disagree	disagree	neutral	agree	strongly agree
1. Safe distance	□	□	□	□	□
2. Keep awareness	□	□	□	□	□
3. Protection strategy	□	□	□	□	□
4. Remove dangerous materials	□	□	□	□	□
5. Other:___________					

Security

	strongly disagree	disagree	neutral	agree	strongly agree
1. Reporting system	□	□	□	□	□
2. Video recording while on duty	□	□	□	□	□
3. Sufficient equipment for protection	□	□	□	□	□
4. Having policepersons escort	□	□	□	□	□
5. Other:___________					

## Appendix B

**Table A1:** Bivariate analysis of reporting to the superiors when emergency medical technicians encounter verbal (N = 113) or physical (N = 75) violence at work.

	Reporting Verbal Violence (N = 10)	Reporting Physical Violence (N = 17)
	ODs (95% CIs)	ODs (95% CIs)
**Age (years)**		
20–34	Ref.	Ref.
35–44	0.74 (0.17–3.32)	0.85 (0.22–3.31)
45–55	2.15 (0.31–14.94)	4.25 (0.61–29.45)
**Working years**		
≤10	Ref.	Ref.
11–15	1.50 (0.15–15.46)	0.27 (0.05–1.50)
≥16	2.20 (0.25–19.59)	0.56 (0.13–2.42)
**Education level**		
2-year college	Ref.	Ref.
4-year college	2.24 (0.44–11.47)	0.70 (0.17–2.88)
Master’s degree	0.76 (0.06–8.94)	1.27 (0.26–6.27)
**EMS deployments per month**		
≤20	Ref.	Ref.
21–50	0.36 (0.04–3.42)	1.38 (0.31–6.26)
51–100	0.78 (0.13–4.62)	0.58 (0.10–3.48)
≥101	1.50 (0.30–7.43)	1.04 (0.21–5.20)

Note: Gender was not included in the analysis due to limited numbers. Abbreviations: ODs, odds ratios.

## Appendix C

**Table A2:** Strategies to prevent workplace violence in emergency medical services systems.

	Verbal Assaults N = 152 (%)	Physical Assaults N = 152 (%)	*p*-Value
**Knowledge and training**			
Educational lectures	82 (53.9)	103 (67.8)	0.014
Simulation training	88 (57.9)	104 (68.4)	0.057
Emotion management consultation	99 (65.1)	108 (71.1)	0.268
Antiviolence law enforcement	114 (75.0)	108 (71.1)	0.438
**Equipment and resource**			
Chemical or physical restraints	89 (58.6)	106 (69.7)	0.042
Video-recording devices	127 (83.6)	128 (84.2)	0.876
Self-defense skills	89 (58.6)	104 (68.4)	0.074
Helmets or bullet-proof vests	87 (52.7)	99 (65.1)	0.157
**System**			
Post-event report system	116 (76.3)	124 (81.6)	0.260
Mutual aid with policepersons	123 (80.9)	127 (83.6)	0.548
Registry of patients with history of violence assaults	119 (78.3)	122 (80.3)	0.671
Registry of substance abuser	116 (76.3)	122 (80.3)	0.404
**Social policy and society**			
Policy of emergency medical services agency	128 (84.2)	125 (82.2)	0.645
Public awareness of workplace violence	115 (75.7)	102 (67.1)	0.990
Standard protocols toward workplace violence	123 (80.9)	122 (80.3)	0.884
Advocating on internet social media	124 (81.6)	122 (80.3)	0.770
**Situational awareness**			
Maintenance of safe distance	135 (88.8)	133 (87.5)	0.722
Improvement of situation sensitivity	135 (88.8)	134 (88.2)	0.857
Early application of protection strategy	121 (79.6)	125 (82.2)	0.559
Removal of dangerous materials	128 (84.2)	129 (84.9)	0.873
**Security**			
Instant reporting and alarming system	119 (78.3)	126 (82.9)	0.310
Real-time video surveillance	133 (87.5)	132 (86.8)	0.863
Sufficient protection equipment	122 (80.3)	121 (79.6)	0.886
Working with police escorts	133 (87.5)	129 (84.9)	0.506
